# Innate and Adaptive Responses to Heat Shock Proteins in Behcet's Disease

**DOI:** 10.1155/2013/249157

**Published:** 2013-12-31

**Authors:** H. Direskeneli

**Affiliations:** Division of Rheumatology, School of Medicine, Marmara University Hospital, Marmara University, Fevzi Çakmak Mah, Mimar Sinan Cadde No. 41, Pendik, 34890 Istanbul, Turkey

## Abstract

Behcet's disease (BD) is a systemic, chronic inflammatory disorder with both innate and adaptive immune responses. Heat shock proteins (HSP) are highly conserved molecules in different species with scavenger activity and involved in correct folding of newly synthesized proteins. T and B cell responses against HSPs are observed in BD patients in both *αβ* and *γδ* T-cell populations. 60-kD HSP (HSP60) is also shown to be recognized by pattern recognition receptors such as toll-like receptors (TLR) and is suggested to be an endogenous “danger” signal to the immune system with rapid inflammatory cytokine releases and enhancement of adaptive Th1-type responses. Elucidating the exact role of HSPs in BD pathogenesis might pave the way to less toxic therapeutic approaches to BD, such as antibacterial therapies and immunomodulation.

## 1. Introduction

Behcet's disease (BD) is a systemic, chronic inflammatory disorder with a diverse spectrum of clinical manifestations including mucocutaneous, ocular, vascular, gastrointestinal, musculoskeletal, and central nervous system involvement [1, 2]. A complex genetic background leading to a proinflammatory, innate-immune system derived activation perpetuated by adaptive immune responses against environmental and autoantigens is accepted as the main pathogenic mechanism in BD [3, 4].

Microbial infection has been implicated in the development of BD since its initial description in 1937 by Hulusi Behcet. Four principal hypotheses have been suggested: (i) bacterial, with *Streptococci* in the foreground, (ii) viral, (iii) indirectly via heat shock proteins (HSP), and (iv) crossreactive or molecular mimicry etiologies [5].

Clinical observations such as increased oral manifestations after dental manipulations, streptococcal hypersensitivity in skin tests, dominance of atypical streptococcus species in BD patients' oral flora, and recent reports of beneficial antibacterial therapy put forward the role of *Streptococcia* in BD [2, 6–8]. As a wide variety of *Streptococcia* (*sanguis, salivarius,* etc.) are implicated, antigens common to various species are logical candidates of immune stimuli in BD [9].

## 2. Heat Shock Proteins: Adaptive Responses 

Heat-shock proteins are a group of intracellular proteins which have scavenger roles for other intracellular proteins under denaturating stress conditions such as infections, hypoxia, trauma, and toxic drugs [10, 11]. Significant sequence homology exists between the mammalian and microbial HSPs (mycobacterial and streptococcal HSP65s have over 90% and human HSP60 over 50% homology) [6], shown recently also with bioinformatic approaches [12]. In addition to their physiological roles, they are implicated in the pathogenesis of various immune-mediated disorders such as infections (tuberculosis and chlamydia), autoimmune diseases (rheumatoid arthritis and multiple sclerosis), vascular thrombosis (atherosclerosis), and malignant disorders [13].

HSP60 with a molecular mass of 60 kD is mainly expressed in mitochondria. However, during stress, an intracellular redistribution of HSP60 and cell surface expression is reported. HSP65 is also expressed on monocytes after IFN-*γ* stimulation and on T-cells going apoptosis [14]. Local HSP60 overexpression is present in oral ulcers of both patients with recurrent oral ulcer patients and BD [15]. Similarly HSP was present more in BD in the epidermal regions of active skin lesions such as erythema nodosum and papulopustules [16]. Increased expression is also observed in intestinal BD lesions [17]. Serum levels of HSP60 were investigated in one study and was higher in BD; however, its level did not correlate with disease activity [18].

A *molecular-mimicry* based pathogenic mechanism for HSPs in BD is first suggested by Lehner et al. that human HSP-responsive T-cells stimulated by microbial counterparts (*cross-reactivity*) might trigger T-cell activation and memory responses [6]. First supporting evidence for this hypothesis is the identification of anti-HSP65 antibodies cross-reactive with oral mucosal homogenates and oral *Streptococcia* [19]. Four epitopes of mycobacterial HSP65 (amino acid sequences 111-25, 154-72, 219-33, and 311-26) and their human counterparts with 50–80% homology were recognized to be immunodominant antigens for T-and B-cell responses in BD in studies from UK, Japan, and Turkey [20–23]. PPD and HSP65 specific long-term T-cell lines (mainly TCR *αβ*+CD4+ or CD8+) are also highly reactive to human HSP60-derived peptides in both BD patients and healthy controls showing that these self-reactive T-cells are escaping central tolerance and are present in the peripheral repertoire [24]. However, most PPD-stimulated lines responded to epitope 425-41 of HSP60 in BD patients (an epitope not described in primary cultures), whereas epitope 336-51 dominated in controls. The reaction pattern changes with HSP60 stimulation, which drives a dominant 336-51 response in both groups. This observation suggested that differential epitope recognition of the immune system associated with the balance of microbial versus human HSP expressions might determine the level of pathogenic self-reactivity in BD.

Although some “*in vitro*” data implicating a Th2 activation is reported, as most other vasculitides, BD is mainly a Th1/Th17 type disorder with interleukin-2 (IL-2), IL-12, interferon-*γ* (IFN-*γ*), and IL-17 cytokine profile. In this context, stimulation of peripheral blood mononuclear cells (PBMC) with human HSP60 peptide 336-51 produced IFN-*γ*, tumor-necrosis factor-*α* (TNF-*α*), and IL-12, whereas Th2 cytokines IL-4 and IL-10 suppressed the proliferative responses in BD [17, 25].

## 3. *γδ*-T-Cells and HSPs 

*γδ* T-cells are a minor T-cell population (1–10% of PB T-cells) that express T-cell receptors (TCRs) comprised of *γ* and *δ* heterodimers [26]. V*γ*9*δ*2+ T-cells, a major subset of *γδ* T-cells in the PBMCs, recognize nonpeptide antigens produced by bacteria. *γδ* T-cells have important roles in immunity as a “*first line of defence*” against microorganisms, surveillance against tumors, and possibly in modulating autoimmune responses [27]. Whereas B cells and *αβ* T-cells are commonly thought to contribute primarily to the antigen-specific effector and memory phases of immunity, *γδ* T-cells are distinct in that they combine conventional adaptive features (inherent in their T-cell receptors and pleiotropic effector functions) with rapid, innate-like responses [28].

Peripheral blood *γδ* T-cells are observed to be elevated in most, but not all studies in BD [29–32]. These *γδ* T-cells are associated with active disease and have higher expression of CD29, CD69, and production of IFN-*γ* and TNF*α* [33]. Whereas PB *γδ* T-cells are mainly V*δ*2+, local fluids such as bronchoalveolar lavage and cerebrospinal fluid are dominated by V*δ*1+ T-cells. Maybe more significant is the local *γδ* T-cell presence in active BD lesions where HSP65 expression is upregulated, with possible HSP-*γδ* T-cell interactions [16].

*γδ*-T-cell activation is also shown with oral flora extracts which might contain HSPs as antigens [30]. HSP-derived peptide responsive T-cells were mainly of *γδ* T-cell subset in UK, whereas CD4+ T-cells are reported from Japan and Turkey [34]. However, in contrast to these data, no response to HSP60 is observed in any T-cell line derived from intraocular fluid of uveitis patients with BD, whereas nonpeptide prenyl pyrophosphate reactive *γδ* T-cells were present [35].

## 4. HSPs and Antibody Responses

Similar to T-cell studies, “*cross-reactivity*” is also demonstrated for anti-HSP60 antibodies. Both antistreptococcal and antiretinal HSP60 antibodies are elevated in BD patients' sera with uveitis [36]. With competitive ELISA, both antigens inhibit the binding of anti-HSP60 antibodies to each other. Increased anti-HSP65 antibody responses are also present in the cerebrospinal fluid (CSF) of neuro-BD patients with parenchymal involvement [37]. Similarly, optical densities obtained from ELISAs against the recombinant human hnRNP-A2/B1, which is shown to be expressed in endothelial cells and is a target antigen of anti-endothelial cell antibodies (AECA) in BD, correlated with those against the recombinant streptococcal hsp65 [38].

## 5. Animal Models

In an animal model with subcutaneous HSP inoculation, human HSP derived, immunodominant peptides caused an experimental uveitis without other symptoms of BD in rats [39]. Oral administration of peptides also induced uveitis in contrast to most models of “*oral tolerance*” where mucosal immune encounter with pathogenic antigens suppress the immune activity. Heat-shock to oral mucosa also increases *S. sanguis* colonisation, oral inflammatory cytokine expressions (IL-2, IL-6, IFN-*γ*, and TNF-*α*), and mild iridocyclitis in mice, implying that stress might be crucial for the breakdown of mucosal defences and anti-HSP reactivity [40].

## 6. Other HSPs and BD

*α*B-crystallin is a small stress protein constitutively abundant in vertebrate eye lens and found in several other organs including skeletal muscle, kidney epithelial cells, and glia cells of central nervous system [41]. Serum and CSF IgG and serum IgM antibody responses to *α*B-crystallin are shown to be elevated in neuro-BD patients. When responses were subclassified according to the type of neuro-BD, similar to anti-HSP65 responses, patients with parenchymal neuro-BD had higher CSF IgG responses to *α*B-crystallin compared to neuro-BD group with intracranial hypertension (vascular involvement). CSF IgG responses to HSP65 and *α*B-crystallin showed a significant correlation with each other, possibly due to similar immune mechanisms driving both autoantibody responses in the CSF. Another recent study, screening with a protein macroarray also led to the identification of stress-induced-phosphoprotein-1 (STIP-1) as an antigenic target for antineuronal antibodies in BD [42].

Elevated anti-HSP70 antibody levels are also observed in patients with BD [43, 44], but not in all studies [45]. However, when free serum HSP70 levels are investigated in the same samples, no correlation is observed between free serum HSP70 and anti-HSP70 antibodies [44, 45]. This observation points to an important difficulty in HSP hypothesis: the role of HSPs in tissue selectivity. HSPs are expressed by all cells under suitable stress conditions, whereas BD involves a limited number of tissues. This selectivity can be explained by differences in local HSP expressions (not reflected in PB), such as preferential HSP expression of the skin and retina.

## 7. Pattern Recognition Receptors and HSPs: Activation of the Innate System Directly 

With its autoinflammatory features, innate immune activation through pattern recognition receptors, NODs, and inflammasome-associated mechanisms are implicated in BD pathogenesis [46, 47]. In addition to being processed and presented to *αβ* and *γδ* T-cells by monocyte-macrophages and stimulating classical, adaptive T-cell responses, HSPs might also activate innate immune mechanisms directly in BD. Recent studies have suggested that HSP60 serves as a “*danger signal*” to the innate immune system [48]. Macrophages, endothelial, and smooth muscle cells were found to elicit a proinflammatory response when incubated with HSP60, releasing IL-6, IL-12, IL-15, and TNF-*α* and upregulating adhesion molecule expressions such as E-selectin, VCAM-1, and ICAM-1 [49]. The proinflammatory response to HSP60 is similar in kinetics and extent to lipopolysaccharide (LPS) stimulation. In early studies, HSP60 is shown to activate mononuclear cells through CD14 which is a high affinity receptor of bacterial LPS on cell membranes. However, later on CD14 is shown to be a coreceptor for a novel molecule of innate immunity, toll-like receptor-4 (TLR4), activating p38 mitogen-activated protein kinase and NF-*κ*B [50]. TLRs are evolutionarily conserved, germline encoded receptors that recognize specific molecular patterns associated with microorganisms [51]. There are currently 13 known TLR members with ligands representing unique products of microbial metabolism such as LPS, peptidoglycan, flagellin, or hypomethylated CpG DNA motifs. Activation of the toll system is suggested to induce dendritic cell (DC) maturation, causing elevated major Histocompatibility Complex (MHC) and costimulatory molecules (CD80 and CD86). Expression of various cytokines such as IL-12, which direct Th1 differentiation by DCs are also associated with TLR signaling. HSP60 is one of the first autoantigens shown to activate the toll system through TLR2 and TLR4 [52]. HSPs released from necrotic (*but not apoptotic*) cells are observed to activate DCs, and HSP60 is shown to induce DC maturation with increased MHC class II, CD40, CD54, and CD86 expressions and allogeneic T-cell proliferation with a Th1 bias [53]. HSP60 is also found to rapidly activate the mitogen-activated protein kinases p38, c-Jun N-terminal kinase, extracellular signal-regulated kinase, and NF-*κ*B in DC. These data support a new model of immunity depending on “*danger*” signals such as HSP60, postulated by Matzinger who suggests that the immune system mainly responds to substances that cause “*damage*,” rather than the classical theory of those that are simply “*foreign*” [54].

In PBMNC analysis, TLR4 expression is shown to be increased in BD patients [55]. In this study, TLR4 levels negatively correlated with heme oxygenase HO-1 (an inducible heme-degrading enzyme that is induced by various stresses) which suppress inflammatory responses. Monocytes of active BD patients also showed higher expressions of TLR2 and TLR4 in PBMNC analysis [56]. *In vitro* analysis, in this study, showed that vitamin D(3) dose-dependently suppress the protein and mRNA expressions of TLR2 and TLR4. In contrast to these studies, TLR6 expressing granulocytes in BD patients was significantly decreased, which enhanced after stimulation with HSP60 and streptococcal extracts [57]. TLR2 and 4 mRNA are also shown to be increased in the intestinal lesions of BD patients and colocalize with HSP60 and IL-12 suggesting that HSP60 may activate Th1 cells through TLRs [58]. Recent genetic data may also implicate the role of TLRs in BD pathogenesis. Genetic associations with TLR2 and TLR4 are shown in some but not all studies [59–62]. A recent, most comprehensive study from China with >800 patients confirmed the role of TLR2 polymorphisms in ocular BD [63]. A recent large study also demonstrated that rare, low-frequency nonsynonymous variants of TLR4 are shown to be increased in BD patients [64].

## 8. HSPs and Other Immune Mechanisms

A final possible role of HPSs is their adjuvant function. In addition to self-presentation discussed previously, HSPs, as molecular chaperones, might transfer antigenic peptides to “*professional*” APCs which then activate specific T-cells or enhance the presentation of MHC-peptide complexes by poorly immunogenic tumor cells. Deficiencies in HLA class I expression on tumor cells are proposed as a mechanism to interfere with the antitumor cytotoxic T-cell responses (CTL). HSP65 transfected clones of melanoma cell lines exhibit significantly increased levels of HLA class I expression and are effectively lysed by alloreactive CTL [65]. Similarly, increased HSP60 expression of APCs may help antigen presentation by BD-associated HLA-class I molecule HLA-B51 to the effector T-cells and enhance pathogenic immune responses. Although an association of anti-HSP60 responses and HLA-B51 is not previously demonstrated, HSP-HLA interactions require further studies.

It was also demonstrated that both HSP65 and HSP70 upregulate CD8+ T-cell derived *β*-chemokine expressions (RANTES, MIP-1*α*, and MIP-1*β*) both directly and also as an adjuvant linked to peptides indirectly [66]. This stimulation of innate immunity might drive adaptive responses and attract APCs (dendritic cells and macrophages) and effector T-cells.

## 9. Specificity of Anti-HSP Responses

T and B cell responses against HSPs are observed in diverse inflammatory disorders. Whether these responses are specific to different disorders or are present as a part of a nonspecific autoimmunity is currently unknown. Some studies (other than BD) using peptide epitopes suggest that adaptive responses can be specific against different T and B cell epitopes. A cross-reactive antimycobacterial HSP65 peptide (aa 91-105) is shown to be specific to recurrent oral ulcer patients compared to HC [67]. Tolerogenic peptides of human HSP60 are also reported only in juvenile idiopathic arthritis cases but not in healthy or diseased controls [68].

## 10. Possible Therapeutic Approaches

Elucidating the exact role of HSPs in BD pathogenesis might pave the way to less toxic therapeutic approaches with HSPs. Immunomodulation with HSPs is demonstrated by “*oral tolerisation*” with peptide 336-51 linked to cholera toxin B subunit, first in an animal model and later in uveitis patients [13, 69]. Similarly, treatments aiming to suppress oral colonization with *Streptococci* leading to less bacterial HSP load might also be effective as adjuvant therapies to immunosuppressives and deserve further studies [70]. Other possible mechanisms of HSP-associated therapeutic approaches may be RNA interference [71], HSP inhibition with synthetic inhibitors [72], inhibition of HSP-ligand interactions [73], or antisense oligonucleotides targeting HSPs [74].
